# Anti-Cancer Potential of Phytochemicals: The Regulation of the Epithelial-Mesenchymal Transition

**DOI:** 10.3390/molecules28135069

**Published:** 2023-06-28

**Authors:** Shuangyu Liu, Lingyu Li, Dongmei Ren

**Affiliations:** Key Laboratory of Chemical Biology (Ministry of Education), School of Pharmaceutical Sciences, Shandong University, 44 West Wenhua Road, Jinan 250012, China; liusy00219@163.com (S.L.); 202121044@mail.sdu.edu.cn (L.L.)

**Keywords:** epithelial-mesenchymal transition, phytochemicals, cancer therapy

## Abstract

A biological process called epithelial-mesenchymal transition (EMT) allows epithelial cells to change into mesenchymal cells and acquire some cancer stem cell properties. EMT contributes significantly to the metastasis, invasion, and development of treatment resistance in cancer cells. Current research has demonstrated that phytochemicals are emerging as a potential source of safe and efficient anti-cancer medications. Phytochemicals could disrupt signaling pathways related to malignant cell metastasis and drug resistance by suppressing or reversing the EMT process. In this review, we briefly describe the pathophysiological properties and the molecular mechanisms of EMT in the progression of cancers, then summarize phytochemicals with diverse structures that could block the EMT process in different types of cancer. Hopefully, these will provide some guidance for future research on phytochemicals targeting EMT.

## 1. Introduction

Cancer has become a significant global public health problem with the continuous aging of the population. Although advancements in the treatment of cancer have been achieved due to the discovery of new targets and technologies, tumor metastasis remains the primary cause of mortality for cancer patients, and more than 90% of cancer-related deaths occur as a result of cancer metastasis [1]. Even if early detection and intervention can greatly increase the survival rate of patients with metastatic cancers, the development of treatments targeting metastatic cancers is still urgent [2].

Epithelial-mesenchymal transition (EMT) is a well-defined, reversible process in which epithelial cells lose their epithelial phenotype and acquire mesenchymal-like features [3]. The origin of EMT is associated with the loss of apical-basal polarity in epithelial cells. During the EMT program, epithelial cells gradually lose their cell-cell contacts due to the disassembly and deconstruction of cell junctions, and then they gain certain characteristics of mesenchymal cells [4]. These mesenchymal cells display enhanced migratory ability and resistance to cell death signals. EMT is a necessary physiological process in embryonic development, tissue repair, and cellular stemness maintenance. However, in a pathological process, improperly regulated EMT is hijacked by cancer cells and plays an important role in carcinogenesis and fibrosis [5,6]. Cancer cells undergoing EMT change in both morphology and motion, accompanied by increased invasion and metastasis potential as well as therapy resistance [7]. Therefore, EMT has emerged as a prospective target for the treatment of cancer in recent years. Preventing the EMT process of cancer cells has become an attractive strategy in cancer therapy.

Some phytochemicals, especially those that originate from plants, have been demonstrated as EMT modulators targeting multiple stages of the process. As a consequence, these phytochemicals provide advantages for controlling cancer cells spreading throughout the body and overcoming treatment resistance. This makes phytochemicals valuable candidates for novel anti-cancer drugs. There have been several review articles published about phytochemicals exerting anticancer effects through inhibiting EMT [8,9,10]. Considering the continuous attractiveness of this topic for researchers, we provide an updated review article here. In this review, we summarize EMT in cancer progression and highlight the implications of phytochemicals in cancer treatment through EMT regulation.

## 2. The Regulation of the EMT Process and Its Roles in Cancer Progression

### 2.1. The Regulation of the EMT Process

EMT is a dynamic process in which epithelial cells go through multiple biochemical changes leading to their conversion into a mesenchymal phenotype [11]. During this process, cells undergo morphological changes associated with the repression of epithelial markers and the acquirement of mesenchymal marker proteins. The most notable marker of epithelial cells is E-cadherin, while N-cadherin is the most representative marker of mesenchymal-type cells [12]. The switch from E-cadherin to N-cadherin is often used for the identification of EMT processes. Downregulation of E-cadherin destabilizes adherens junctions, resulting in a loss of affinity for epithelial cells; upregulation of N-cadherin mediates a greater affinity for mesenchymal cells. Other epithelial markers, including claudins and occludins, and mesenchymal markers, including vimentin and fibronectin, participate in altering cell-cell affinity together [13]. These markers can also be used for the identification of EMT processes.

The exchange of gene expression from epithelial to mesenchymal phenotypes is initiated by at least four layers of regulation: transcriptional control, small non-coding RNAs, differential splicing, translational control, and post-translational control [14]. Undoubtedly, transcriptional control is the most extensively studied network. EMT-inducing transcription factors (EMT-TFs) are the core of the transcriptional control network of EMT, and multiple signaling pathways lead to the regulation of EMT-TFs.

EMT-TFs directly or indirectly contribute to the regulatory network of the EMT process. Some TFs, including SNAIL1, SNAIL2, ZEB1, ZEB2, E47 (also known as transcription factor-3, TCF-3), kruppel-like factor 8 (KLF8), and Brachyury, repress the expression of E-cadherin through direct binding to the CDH1 promoter, which encodes E-cadherin. Simultaneously, these TFs also repress other junctional proteins, such as claudins. Some other TFs, including TWIST1, hepatocyte nuclear factor 3 (FOXC2), gooseciod, E2-2, SIX1, and paired mesoderm homeobox protein 1 (PRRX1), regulate the EMT process without direct binding to the CDH1 promoter. Among these TFs, some are very common in most studies about EMT, particularly the nuclear factors of the SNAIL, ZEB, and TWIST families. This collection of TFs seems to be enough for the regulation of EMT [15]. Most of the other TFs, which have only been mentioned in a few studies, may just have an assisting role [16].

A variety of signaling pathways collaboratively regulate EMT progression, mainly including the transforming growth factor β (TGF-β), wingless-type MMTV integration site family (Wnt), Hedgehog, and epidermal growth factor receptor (EGFR) pathways. Some other signaling pathways, such as Notch, Hippo, and nuclear factor kappa-B (NF-κB), also regulate EMT in certain kinds of cancer. All of these pathways eventually converge at the level of transcription factors such as ZEB, SNAIL, and TWIST to regulate the EMT process [11].

**Figure 1:**
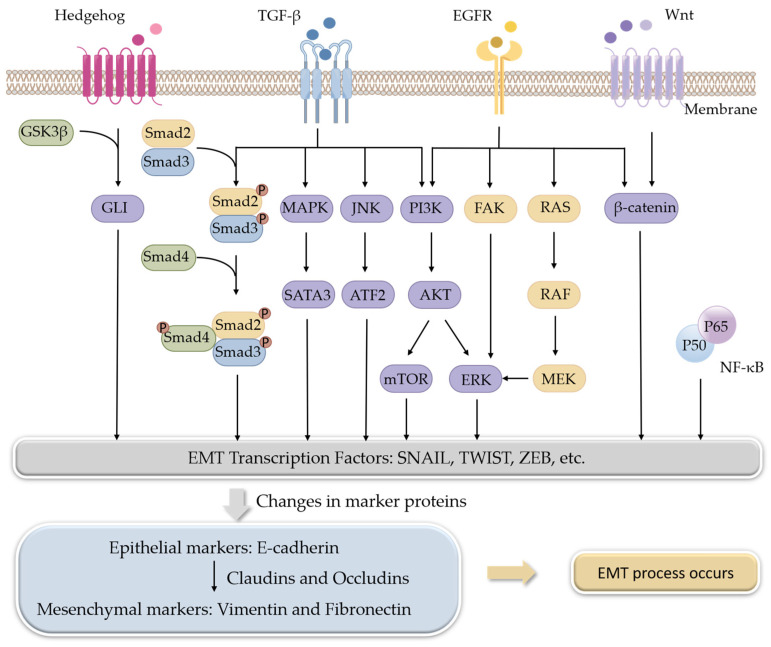
Signaling pathways involved in the regulation of EMT.

TGF-β, a multifunctional cytokine, is the best-known EMT inducer. When TGF-β ligands bind to TGF-β receptors, signals are transmitted into cells through its intracellular transducers, Smads, or factors other than Smads, such as phosphoinositide 3-kinase (PI3K), mitogen-activated protein kinases (MAPKs), and c-Jun N-terminal kinase (JNK). Both the Smads-dependent and Smads-independent pathways are involved in the regulation of EMT [17,18]. The Wnt signaling pathway is another multifunctional pathway to induce EMT. β-Catenin is the downstream factor of the Wnt receptor. The Wnt signaling pathway induces EMT through the interaction of β-catenin with some transcription factors [19]. The hedgehog signaling pathway exerts its role by terminating at a transcription factor, glioma-associated oncogenes (GLI), and aberrant activation of Hedgehog/GLI induces EMT [20,21]. EGF is also a well-known EMT inducer. Through binding to its receptor EGFR, EGF-induced signals are transmitted by PI3K, focal adhesion kinase (FAK), or rat sarcoma (RAS) pathways [22,23] (Figure 1).

### 2.2. EMT Modulates Cancer Progression

Because abnormal activation of EMT gets involved in many stages of cancer progression, it is easy to conclude that the pathways and molecular targets of EMT are related to the poor prognosis of cancers. Generally, metastasis and chemotherapeutic resistance in cancer are major consequences of EMT activation.

**Figure 2:**
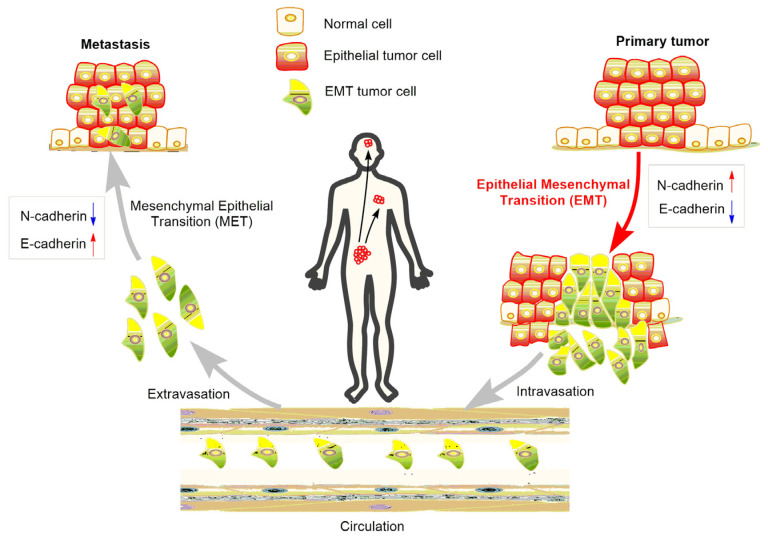
Role of EMT in the metastasis of cancer progression.

At the initial stage of EMT, malignant epithelial cells in the primary tumor obtain the ability to lose their cell-cell connections and detach from the primary tumor. The detached cells or cell clusters acquire characteristics of mesenchymal-like cells, break through the basement membrane, invade surrounding tissue, and gain access to blood vessels to achieve spread and dissemination [24]. The acquired mesenchymal properties assist the survival of cancer cells in the circulation and, in addition, provide resistance to cell death signals. When the circulating tumor cells arrive in a distant position of the body with a suitable micro-environment, the spreading cells pass through the blood vessels again and colonize to form metastases [25] (Figure 2). As a consequence, suppressing EMT in cancer cells or developing anti-EMT adjuvants emerge as attractive strategies for cancer treatment.

## 3. Phytochemicals with the Effects of Interfering EMT

As mentioned above, the complex EMT process is mediated by diverse cell signaling pathways, and whichever molecules can interfere with elements of these signaling pathways that are involved in the progression of EMT will be a good choice for developing anti-EMT candidates. Anti-EMT will provide advantages for the inhibition of cancer metastasis or treatment resistance. Phytochemicals have long been recognized as a resource for anti-cancer drugs. In recent years, some phytochemicals have been found to exert anti-cancer effects by inhibiting or reversing the EMT process. The diverse structures allow phytochemicals to interfere with multifaceted EMT-related targets. Here we list some major EMT-modulating phytochemicals according to their structural classification and summarize their functions in cancer treatment.

### 3.1. Phenylpropanoid

**Figure 3:**
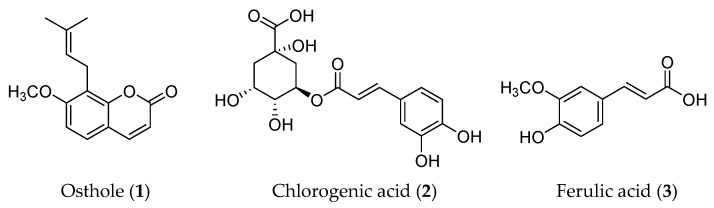
Structures of phenylpropanoids with EMT inhibitory effects.

Phenylpropanoid is a class of naturally occurring compounds with a C6-C3 skeleton. Based on the number of C6-C3 units and the structure, phenylpropanoids can be divided into three categories: simple phenylpropanoids, coumarins, and lignans. Phenylpropanoids are reported to possess a wide range of bioactivities, including anti-tumor, anti-inflammation, neuroprotection, osteogenic effects, cardiovascular protection, anti-bacterial, and anti-parasitic effects [26]. Three phytochemicals in this type of compound, osthole (**1**), chlorogenic acid (**2**), and ferulic acid (**3**), have been proven to have anti-cancer effects by regulating the EMT process (Figure 3).

#### 3.1.1. Osthole (**1**)

Osthole (**1**) is a coumarin that is mainly isolated from *Cnidium monnieric* [27]. This compound has been shown to induce a range of beneficial bioactivities, including anti-cancer, anti-epileptic, and anti-inflammatory properties [28]. In terms of the anti-cancer effects of osthole, it could inhibit proliferation, induce apoptosis, avoid invasion and migration, prevent angiogenesis, and increase chemosensitivity [29]. The current research on osthole indicates that its anti-cancer effects in renal carcinoma cells, brain cancer cells, and liver cancer cells are involved with EMT suppression. In renal cancer 786-O and ACHN cells, osthole pre-treatment decreased the migration and invasion of renal cancer cells dose-dependently. Further exploration indicated that epithelial biomarkers (E-cadherin and β-catenin) increased while mesenchymal biomarkers (N-cadherin and vimentin) decreased. Meanwhile, the downregulation of Smad-3, SNAIL1, and TWIST-1 suggested that osthole suppressed EMT through the inhibition of EMT transcription factors [30].

Insulin-like growth factor-1 (IGF-1) was able to induce EMT in brain cancer cells, which contributes to the proliferation and migration of glioblastoma multiforme, a kind of aggressive brain tumor. In an IGF-1-treated GBM8401 cell model, osthole reversed IGF-1-induced cell morphological changes, increased the level of epithelial biomarkers, and decreased the level of mesenchymal biomarkers, demonstrating that osthole could inhibit the IGF-1-induced EMT process. Further investigation indicated that osthole suppressed IGF-1-induced EMT at the transcriptional level. The inhibition of the PI3K/pAKT signaling pathway is involved in osthole-inhibited IGF-1-mediated EMT [31]. In four kinds of liver cancer cell lines, osthole inhibited cell proliferation and migration and also inhibited EMT, as evidenced by the enhanced expression of epithelial biomarkers and reduced expression of mesenchymal biomarkers [32].

#### 3.1.2. Chlorogenic Acid (**2**)

Chlorogenic acid (CGA, **2**) is an ester of caffeic acid and quinic acid and is the major constituent of the Chinese herbal medicine *Lonicerae japonicae* [33]. Usually, CGA is considered a nutraceutical with low cytotoxicity; most studies are mainly concerned with its beneficial effects for health, such as anti-oxidant, anti-hypertension, anti-obesity, and anti-diabetic effects [34,35,36]. There is only a small amount of research focused on the anti-cancer effects of CGA and its metabolites or derivatives. There is a study reporting that CGA induced apoptosis in four kinds of breast cancer cells and retarded tumor growth in mice bearing 4T1 cell xenografts. The results of a wound-healing assay and a transwell assay proved that CGA inhibited metastasis and invasion of breast cancer cells. The western blotting and immunohistochemistry results indicated a change in E-cadherin and N-cadherin, suggesting that an EMT suppression occurred [37]. In another study, more evidence was provided to demonstrate that CGA reversed the EMT process by binding directly to lipoprotein receptor-related protein 6 (LRP6) in breast cancer cells [38]. In triple-negative breast cancer MDA-MB-231 cells, a derivative of CGA, isochlorogenic acid C (ICAC), did not affect cell growth but reduced invasion and migration significantly. These roles were further demonstrated to be mediated by the inhibition of the EGFR-induced EMT process [39].

#### 3.1.3. Ferulic Acid (**3**)

Ferulic acid (FA, **3**) is a simple phenylpropanoid contained in several commonly used Chinese herbal medicines, such as *Ferula sinkiangensis*, *Angelica sinensis*, and *Ligusticum chuanxiong*. Similar to CGA, FA was usually regarded as a low-toxic anti-oxidant, and most of its beneficial effects, including anti-inflammation, anti-cancer, and anti-fibrotic effects, were considered to be related to its anti-oxidant properties [40]. FA was revealed to induce apoptosis and inhibit migration using a mesenchymal-like breast cancer cell line, MDA-MB-231. Flow cytometry, wound healing, and transwell assays were used for the detection of the effects exerted by FA in MDA-MB-231 cells, and a xenograft model was used for the in vivo assay. Moreover, EMT was shown to be suppressed in FA-treated MDA-MB-231 cells, as evidenced by the changes in protein and mRNA levels of EMT biomarkers; this was considered to be related to the anti-metastasis effect of FA [41]. In terms of EMT inhibition induced by FA, another study reported its protective effect. In an animal model of silica-induced pulmonary fibrosis, FA halted the progression of pulmonary fibrosis, ameliorated the expression of fibrotic proteins, and prevented EMT. TGF-β/Smad signaling, which is a significant contributor to EMT, was found to be inhibited by FA [42].

### 3.2. Flavonoids

**Figure 4:**
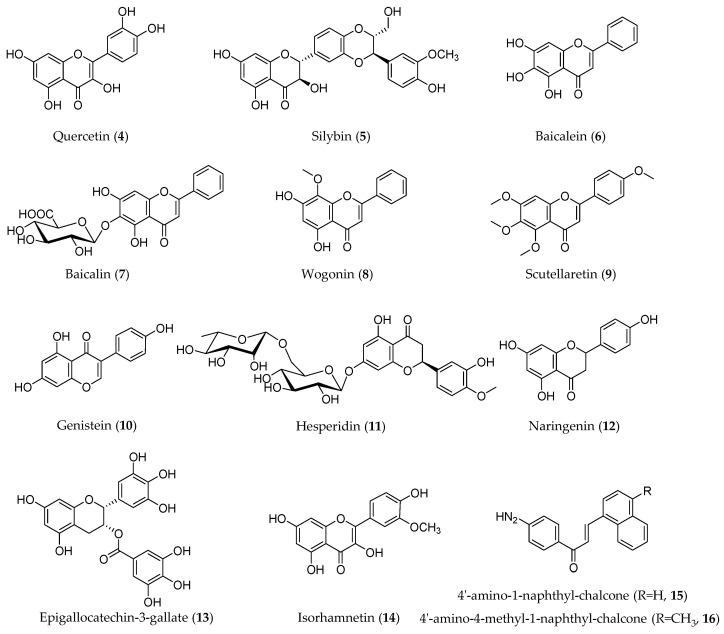
Structures of flavonoids with an EMT inhibitory effect.

Flavonoids are compounds with a skeletal structure of C6-C3-C6, that is, two aromatic rings connected by a three-carbon bridge. Due to the different patterns of the three carbon bridges, flavonoids can be divided into several categories, including flavones, flavonols, flavanones, flavanonols, anthocyanins, flavan-3-ols, isoflavones, chalcones, and xanthones [43]. Flavonoids have a variety of positive health effects, such as anti-bacterial, anti-cancer, anti-osteoporosis, and anti-viral functions [44]. In this type of compound, quercetin (**4**), silybin (**5**), baicalein (**6**), genistein (**10**), hesperidin (**11**), and naringenin (**12**) have been reported to have EMT inhibitory effects (Figure 4).

#### 3.2.1. Quercetin (**4**)

A dietary flavonoid called quercetin (**4**) can be found in a variety of fruits, vegetables, and grains. It possesses anti-inflammatory, anti-cancer, and anti-oxidant properties and could inhibit the growth of several kinds of cancer cells [45,46]. There is a certain amount of research about quercetin-induced EMT reversion. In two glioblastoma cell lines, U87 MG and CHG5, quercetin markedly decreased cell growth, migration, and invasion, as demonstrated by the results of MTT, wound healing, and transwell assays. In a U87 MG cell xenograft mouse model, quercetin also suppressed tumor growth and mesenchymal transition. Because EMT is closely related to the high invasion ability of glioblastoma, the expression of EMT-related biomarkers was detected, and the results indicated that quercetin affected the EMT process in glioblastoma cells. Further investigations revealed that the glycogen synthase kinase 3β (GSK-3β)/β-catenin/ZEB1 signaling pathway, a negative regulator of EMT, was suppressed by quercetin in U87MG and CHG5 cells [47]. In triple-negative breast cancer cells, quercetin reversed the EMT process by blocking the signal transduction of insulin-like growth factors (IGF1)/IGF1 receptor (IGF1R) or modulating β-catenin target genes [48,49]. In the study of prostate cancer, two distinct regulatory systems involved in EMT have been revealed. One is the EGFR/PI3K/AKT pathway [50]; the other one is a long non-coding RNA, MALAT1. This was the first time that MALAT1 was verified to play an important role in quercetin-induced EMT suppression [51]. Additionally, in pancreatic cancer cells, quercetin prevented EMT by obstructing the signal transducer and activator of transcription 3 (STAT3) signaling pathway, reversed interleukin-6 (IL-6)-induced EMT, and consequently decreased cancer cell invasion [45].

Except for its roles in treating cancer, quercetin-induced EMT also played important roles in the treatment of fibrosis diseases such as renal fibrosis and pulmonary fibrosis. In TGF-β1 treated renal epithelial cells NRK-52E, quercetin inhibited the activation of Hedgehog signaling, ameliorated EMT, and improved renal fibrosis. These effects were also verified in rats with unilateral ureter obstruction [52]. One of the typical toxic effects of bleomycin is the induction of pulmonary fibrosis, and the major cause is EMT of alveolar type II epithelial cells. Quercetin cotreatment with bleomycin in RLE/Abca3 cells, which have an alveolar type II cell-like phenotype, suppressed all of the EMT-related changes induced by bleomycin [53].

Quercetin can also be used for chemotherapy medications as an adjuvant due to its EMT reversal effect. It has been reported that quercetin could combat the acquired chemoresistance of erlotinib and cisplatin. Using two established erlotinib-resistant oral squamous cell lines, ERL-R5 and ERL-R10, quercetin was proven to effectively inhibit cell growth by MTT and colony formation assays. This anti-cancer effect was further confirmed in mice bearing ERL-R5 cells. Lowering the expression of PKM2 contributed to the sensitivity of quercetin to erlotinib. Further, PKM2 siRNA cotreatment with erlotinib resulted in further reduction of EMT, suggesting that PKW2 seemed to be crucial in acquiring resistance to erlotinib [54]. In nasopharyngeal carcinoma 5-8F and C-666-1 cells, quercetin induced cell death and inhibited migration and the EMT process. Quercetin also restored the sensitivity of cisplatin-resistant cells. In vivo studies with cisplatin-resistant 5-8F and C-666-1 cells transplanted into mice gave further proof of the effects of quercetin. Changes in EMT markers detected by western blotting indicated that quercetin treatment induced EMT suppression. The yes-associated protein (YAP) downregulation, which led to the recovery of the Hippo pathway, was supposed to be the mechanism of quercetin in nasopharyngeal carcinoma [55].

#### 3.2.2. Silybin (**5**)

The phytochemical silybin (**5**), also known as silibinin, is isolated from the milk thistle (*Silybum marianum*). Structurally, silybin is a hybrid of flavonoid and lignan, but usually it is classified as a flavolignan. This phytochemical is well-known for its hepatoprotective effect and normally exists in some health products [56]. Silybin also exhibited EMT-inhibiting effects in different cancer cells by regulating multiple molecular targets or pathways. EMT drives acquired resistance to ALK tyrosine kinase inhibitors (ALK-TKIs) in lung cancer cells, while silybin co-exposure re-sensitizes lung cancer cells to ALK-TKIs by targeting the TGF-β/Smad signaling axis [57]. In bladder cancer cells, silybin attenuated TGF-β-induced migration and invasion through inhibiting EMT associated with downregulating COX2 [58]. One more study verified that silybin decreased metastasis in vitro and in vivo using the highly metastatic cell line T24-L. Inactivation of β-catenin/ZEB-1 signaling was supposed to be the mechanism of blocking EMT by silybin in this study [59].

In addition to being a potential therapeutic agent to inhibit EMT, silybin has value in joint therapy to overcome the side effects of cancer therapies. STAT3 activation is a new mechanism of crizotinib resistance that involves immune escape and the EMT pathway. Silybin-based cotreatment targeted STAT3 inhibition together with inhibition of the EMT process in crizotinib-refractory cells, thus reversing acquired resistance and restoring sensitivity [60]. Silybin is also synergistic in radiotherapy. Radiotherapy is frequently utilized in prostate cancer treatment, but radiation increases the invasiveness of surviving radioresistant cancer cells during treatment. Combining silybin with radiation not only decreased proliferation but also strongly reduced prostate cancer cell invasion. Most of the migratory and EMT-promoting actions induced by radiation were decreased by silybin [61].

Besides cancer therapy, silybin showed anti-fibrotic effects in a TGF-β induced in vitro model of fibrosis and radiation-induced intestinal fibrosis. In these two anti-fibrotic processes, EMT inhibition induced by silybin played a certain role [62,63].

#### 3.2.3. Baicalein (**6**)

Baicalein (**6**) is the major constituent of a famous traditional Chinese medicine called *Scutellaria baicalensis*. This phytochemical has a wide range of bioactivities, including anti-cancer effects [64]. In breast cancer MDA-MB-231 cells, baicalein exposure significantly inhibited cell migration and invasion. The EMT process was inhibited correspondingly. Downregulation of Cyr61/AKT/GSK-3β pathway was clarified as the anti-EMT mechanism of baicalein in this study [65]. In cervical cancer HeLa cells, baicalein targeted TGF-β inhibition and showed an EMT-suppressing effect [66]. In colorectal cancer H29 cells, baicalein affected cell mobility and reversed the EMT process; SNAIL expression was thought to play an important role in this study [67].

Following discovery, a series of derivatives of baicalein have shown an EMT-suppressing effect. In non-small cell lung carcinoma cells, the inhibition of EMT by baicalin (**7**) was clarified through the PDK1/AKT signaling pathway [68]. There are two analogues of baicalein from *Scutellaria baicalensis*, wogonin (**8**) and scutellaretin (**9**), which inhibit cell proliferation and metastasis in colon cancer cells and liver cancer cells, respectively. In colon cancer cells, wogonin relieves neoplastic behaviors and EMT through the IRF3-mediated Hippo signaling pathway [69]. In liver cancer cells, scutellaretin regulates PI3K/AKT/NF-κB signaling through PTEN upregulation [70].

There is one report that mentions that baicalein regulates the radiosensitivity of cervical cancer cells. Combining baicalein with X-ray irradiation increased the death rate more than irradiation alone. EMT inhibition was detected simultaneously. Upregulation of miR-183 through the inactivation of the JAK2/STAT3 signaling pathway was considered the mechanism of baicalein in cervical cancer cells [71].

#### 3.2.4. Genistein (**10**)

Genistein (**10**) is a cancer-preventive phytochemical mainly found in soy and other legumes; structurally, it is 4′,5,7-trihydroxy isoflavone [72]. Several studies focused on the EMT inhibition effects of genistein. In hepatocellular carcinoma, genistein restricted cell growth and metastasis by attenuating the EMT process, and upregulation of miR-1275-mediated EIF5A2/PI3K/AKT pathway inhibition led to the suppression of EMT and stemness of cells [73]. In papillary thyroid carcinoma cells, genistein significantly decreased the invasion ability of cells and partially inhibited the EMT process. Knockdown of β-catenin reversed the effect of genistein on EMT, indicating that β-catenin involved in genistein-induced EMT modulation [74]. Genistein can be used in combination with other chemotherapeutic compounds. Trichostatin A is a specific inhibitor of histone deacetylases. In Hep-2 laryngeal cancer cells, genistein treatment alone mildly inhibited cell growth and invasion and, in addition, reversed EMT. By joint use of trichostatin A, the effect of EMT inhibition caused by genistein was further increased [75].

#### 3.2.5. Hesperetin (**11**)

Hesperetin (**11**) is an abundant flavanone in citrus fruits. It has been reported that this phytochemical possesses cellular protective effects against multiple cell damage factors. EMT reversion is usually involved in the regulation mechanism of hesperetin. Because of the relationship between EMT and cancer cell migration, hesperetin has been reported to be used in cancer cells to block invasion and metastasis. In cervical cancer cells, hesperetin inhibited EMT-mediated cell invasion and migration by decreasing abnormal activation of the TGF-β/Smads pathway [76]. Typically, the protective roles of hesperetin were given more attention, such as its anti-fibrosis effects. The potential mechanism study in renal fibrosis indicated that hesperetin inhibited EMT and renal fibrosis in UUO mice and TGF-β1-treated NRK-52E cells through suppression of the Hedgehog signaling pathway [77].

#### 3.2.6. Naringenin (**12**)

With a similar structure to hesperetin, naringenin (**12**) is also a flavanone in citrus fruits. Generally, naringenin is a safe supplement that exerts beneficial roles for human health [78]. Emerging studies have shown the anti-cancer potential of naringenin. In glioblastoma cells, naringenin inhibited invasion and metastasis through multiple mechanisms, including EMT modulation, as evidenced by the alteration of EMT biomarkers [79]. In pancreatic cancer cells, naringenin downregulated EMT markers by inhibiting the TGF-β/Smad3 pathway; consequently, invasiveness and metastasis of cells were decreased [80].

#### 3.2.7. Other Flavonoids

Some flavonoids were reported to modulate the EMT process, but they mainly focused on protective effects other than anti-cancer effects, such as epigallocatechin-3-gallate (EGCG, **13**). This phytochemical is the main component of green tea. There is no doubt that EGCG is good for health. Some toxicants exert toxic effects by promoting the EMT process, while EGCG counteracts this process to provide beneficial effects. A study used cigarette smoke exposure to stimulate prostatic EMT and fibrosis, and then EGCG exerted a strongly anti-fibrosis effect by modulating EMT and downregulating the hedgehog pathway [81]. Some flavonoids were reported for their EMT modulation and anti-cancer effects, but only a small number of such studies were conducted, for example, the EGCG derivative (EGCGD) from dark tea and isorhamnetin. EGCGD synergized with gefitinib through suppression of EMT, and isorhamnetin (**14**) blocked AKT/ERK-mediated EMT in A549 cells, which in turn prevented migration and invasion [82]. Two amino-substituted chalcones (**15**, **16**) were discovered with the roles of suppressing migration and invasion of osteosarcoma cells mediated by p53-regulated EMT-related genes [83].

### 3.3. Non-Flavonoid Polyphenolic Compounds

**Figure 5:**
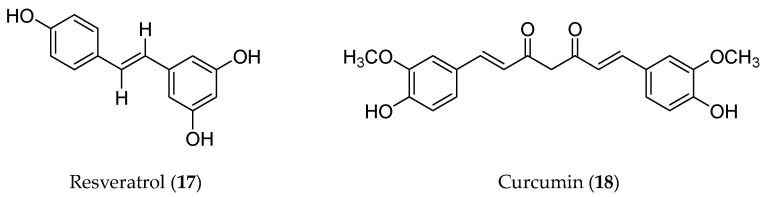
Structures of non-flavonoid polyphenolic compounds with an EMT inhibitory effect.

Polyphenolic compounds refer to a diverse group of phytochemicals containing multiple phenolic functionalities. Flavonoids are the major category of polyphenols. Resveratrol (**17**) and curcumin (**18**) are two well-known compounds that do not belong to flavonoids, so they were listed here as non-flavonoid polyphenolic compounds (Figure 5) to describe their roles in EMT regulation.

#### 3.3.1. Resveratrol (**17**)

Resveratrol (RSV, **17**) is a naturally occurring polyphenol with a range of biological effects good for health [84]. RSV is well recognized as a bioactive substance in red wine and has therefore attracted lots of interest in conducting various studies [85]. Because of the high popularity of RSV, even in such a small research field as EMT, many studies are also emerging. Regarding EMT inhibition, RSV could act on almost all common cancer types, such as colorectal [86], pancreatic [87], gastric [88], prostate [89], lung [90], liver cancer [91], and glioblastoma [92]. The consequences of suppressing EMT in these cancer cells inhibited cell invasion and migration without exception, repeatedly proving the EMT process is crucial for cancer metastasis; moreover, resveratrol provided a strategy to overcome tumor metastasis through EMT impeding.

Different regulation mechanisms were revealed in different cancer cell lines under RSV treatment. The family of microRNAs miR-200 plays an important role in the regulation of the EMT process during metastasis and cancer progression. In RSV-treated colorectal cancer HCT-116 cells, miR-200c expression was upregulated and switched from an EMT to a MET phenotype [93]. In the TNF-β induced colorectal cancer cell EMT model, RSV blocked EMT through suppression of NF-κB and FAK [94]. Another study in colon cancer cells revealed that RSV reversed the EMT process through AKT/GSK-3β/SNAIL signaling [86]. In prostate cancer cells, TNF receptor-related factor 6 (TRAF6) was considered a target of RSV to regulate the EMT process. RSV inhibits EMT progression by repressing the TRAF6/NF-κB/SLUG axis [89]. A study was conducted on glioma stem cells, which were believed to be the driving force of cancer progression. RSV exposure strongly decreased glioma stem cell motility through modulating Wnt signaling [92]. However, in a TGF-β induced glioblastoma EMT model, RSV was shown to suppress EMT through a Smad-dependent pathway [95]. In the TGF-β induced gastric cancer cell EMT model, RSV suppressed EMT through inactivation of Hippo-YAP signaling [96], while in the gastric cancer cell EMT model, but induced by hypoxia, RSV regulated the EMT process via Hedgehog pathway suppression [97]. Nutrition deprivation autophagy factor-1 (NAF-1) is overexpressed in pancreatic cancer cells and tissue and is correlated with cancer invasion. RSV was proven to downregulate the expression of NAF-1 and thus effectively inhibit EMT to exert anti-metastasis roles [84].

RSV also plays a role in some non-tumor diseases by inhibiting the EMT process. EMT is involved in the pathogenesis of endometriosis, a kind of benign disease with some malignant features. Metastasis-associated protein 1 (MTA1) promotes endometriosis by inducing EMT through ZEB2. RSV is effective for the treatment of endometriosis. MTA1 was supposed to be the target of RSV, as evidenced by the decreased expression of MTA1 and suppressed EMT in RSV-treated endometrial cells [98]. Pretreatment with RSV played a protective role in gentamicin-induced nephrotoxicity. Further study revealed that RSV suppressed the EMT process involving TGF-β/Smad pathway to counteract the side effects of gentamicin [99].

#### 3.3.2. Curcumin (**18**)

Curcumin (**18**) is a well-known natural polyphenol derived from the rhizome of *Curcuma longa*. The structure of this compound belongs to diarylheptanoids. Due to its broad bioactivities, including chemoprevention, anti-cancer, and anti-inflammation, curcumin has attracted much interest and has been proven as an EMT suppressor in many kinds of cancer [12]. The benefits of inhibiting the EMT process by curcumin in malignant tumors lie mainly in preventing tumor metastasis.

In different cancer cells, curcumin acts on different potential targets or signaling pathways to regulate the EMT process. Hepatocyte growth factor (HGF) promoted EMT in meningioma, lung cancer, and oral cancer cells. Curcumin treatment blocked the activation of cellular-mesenchymal epithelial transition factor (c-MET), a specific receptor of HGF, and resulted in the inhibition of EMT. In meningioma and lung cancer cells, the c-MET-dependent PI3K/AKT/mTOR signaling pathway has been involved in the mechanism of EMT suppression of curcumin [100,101,102]. In colorectal cancer cells, the upregulated expression of miR-200c and downregulation of its direct target gene, EPM5, were necessary for curcumin-inhibited EMT [103]. TAp63α is a transcription factor that acts as a cancer suppressor gene and, upon overexpression, transcriptionally decreases the expression of miR-19, which consequently inhibits lung cancer EMT. In a tobacco smoke-stimulated lung cancer EMT model, curcumin inhibited EMT by increasing TAp63α expression and decreasing miR-19 levels [104]. In pancreatic stellate cells, curcumin treatment inhibited their migration and secretion of IL-6 under hypoxia. In addition, pancreatic stellate cells in conditioned media modulated pancreatic cancer cells EMT, which was suppressed by curcumin. These indicated that curcumin played an important role in tumor-stromal crosstalk and thus inhibited EMT. IL-6/ERK/NF-κB pathway inhibition was involved in the regulation effect of curcumin [105].

The EMT inhibition of curcumin played a certain role in reversing drug resistance in colon cancer cells. In 5-fluorouracil-resistant HCT116 colon cancer cells, curcumin reversed the resistance by regulating the TET1-NKD-Wnt signaling pathway to inhibit the EMT process [106], while in oxaliplatin-resistant HCT116 colon cancer cells, curcumin overcame the resistance by regulating the TGF-β/Smad2/3 signaling pathway to inhibit the EMT process [107].

### 3.4. Quinones

**Figure 6:**
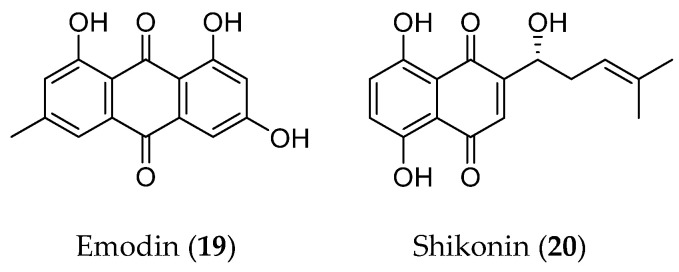
Structure of quinones with an EMT inhibitory effect.

Quinones are compounds that contain intramolecular unsaturated cyclic diketones in their structures. Quinones can be further divided into benzoquinone, naphthoquinone, phenanthraquinone, and anthraquinone. Two well-known phytochemicals, anthraquinone emodin (**19**) and naphthoquinone shikonin (**20**), were demonstrated to regulate the EMT process in several kinds of cancer cells [108] (Figure 6).

#### 3.4.1. Emodin

Emodin is a naturally produced anthraquinone, mainly isolated from *Rheum palmatum* and *Polygonum cuspidatum* [109]. There have been some studies about the regulation of EMT by emodin, mainly focused on two aspects: anti-cancer and anti-fibrosis. Emodin exerts anti-invasion and anti-migration effects by regulating EMT in various kinds of cancer cells through different mechanisms. An EMT regulator, TWIST1, was ectopicly expressed in head and neck carcinoma FaDu cells to trigger EMT and acquire a mesenchymal phenotype. Emodin successfully reversed this process and inhibited TWIST1-induced invasion. The in vivo effect was investigated by the detection of pulmonary colonization by intravenously injected tumor cells. Mechanically, emodin inhibited TWIST1 binding to the E-cadherin promoter and repressed E-cadherin transcription [110]. In ovarian cancer cells, emodin inhibited the EMT process through the ILK/GSK-3β/Slug signaling pathway [111], while in colon cancer cells, emodin inhibited cell invasion and migration by suppressing EMT via the Wnt/β-catenin pathway [112]. In pancreatic cancers, the decreased expression of miR-1271 promoted the occurrence of EMT and metastasis; emodin boosted the expression of miR-1271 and substantially suppressed the EMT process and invasion [113]. In hepatocellular carcinoma cells, emodin treatment activated autophagy and promoted autophagic flux, simultaneously reversed EMT, and indicated the correlation between EMT and autophagy. Both EMT and autophagy regulation were revealed to be attributed to the PI3K/AKT/mTOR pathway [114]. By regulating EMT, emodin could reduce cancer cell resistance to chemotherapeutic drugs, such as gemcitabine resistance in pancreatic cancer cells and doxorubicin resistance in small-cell lung cancer cells [115,116].

#### 3.4.2. Shikonin

Shikonin is a phytochemical with a naphthoquinone skeleton; it is the major constituent of Chinese herbal medicine, *Lithospermum erythrorhizon* [117,118]. Reports on the EMT inhibition of shikonin focused on triple-negative breast cancer cells. According to several reports, shikonin depressed invasion and migration in breast cancer MCF-7, MDA-MB-231, BT549 [119], and 4T1 cells. EMT inhibition was attributed to the anti-metastasis effects of shikonin. Different signaling pathways were getting involved in the regulation of the EMT process, such as NF-κB-Snail signaling in LPS-pretreated MCF-7 and MDA-MB-231 cells [118], the miR-17-5p/PTEN/AKT pathway, and GSK-3β/β-catenin signaling in MDA-MB-231 cells [119,120]. A series of semi-synthesized shikonin derivatives showed anti-proliferation effects in MDA-MB-231 cells; two of them inhibited the EMT process by regulating the PDK1/PDHC pathway [121]. In hepatocellular carcinoma cells, shikonin suppressed the progression and EMT by regulating the miR-106b/SMAD7/TGF-β signaling pathway [122].

### 3.5. Terpenoids

**Figure 7:**
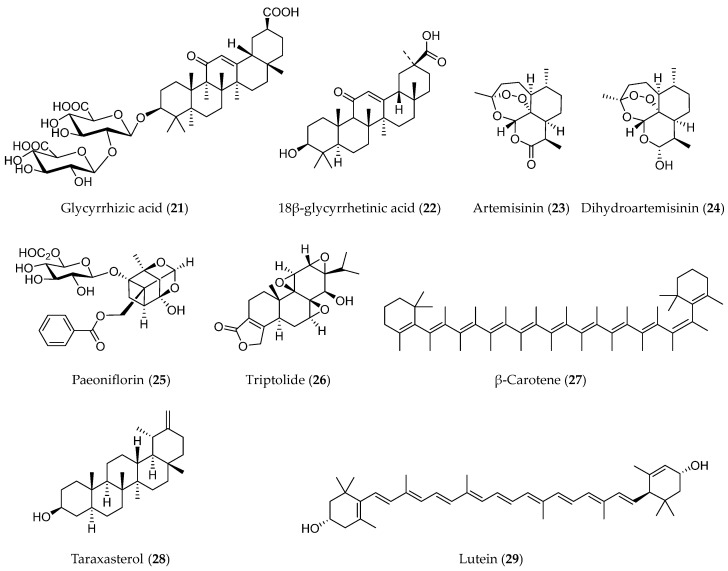
Structures of terpenoids with EMT inhibitory effects.

Terpenoids represent the most numerous and diverse phytochemicals. Structurally, the skeleton of terpenoids contains C-5 isoprenoid units and can be categorized according to the number of isoprenoid units [123]. Terpenoids show a range of bioactivities, such as anti-tumor, anti-inflammatory, anti-bacterial, anti-viral, anti-malarial, and hypoglycemic properties [124]. Several terpenoids, including glycyrrhizic acid (**21**), artemisinin (**23**), paeoniflorin (**25**), triptolide (**26**), and some others (Figure 7), have been proven to be able to suppress tumor progression through regulating the EMT process.

#### 3.5.1. Glycyrrhizic Acid (**21**)

Glycyrrhizic acid (GA, **21**) is the major constituent of *Glycyrrhiza uralensis*, *Glycyrrhiza glabra*, or *Glycyrrhiza inflata*, three species listed under the item *GANCAO* in the Chinese Pharmacopoeia. GA is used as the quality control indicator of *GANCAO* due to its abundant content and diverse bioactivities [125]. GA is a triterpenoid from a structural perspective, and a number of studies have revealed the hepatoprotective, anti-inflammatory, immunostimulatory, anti-viral, and anti-cancer effects of this compound [126,127]. Here, several studies about the inhibition of EMT by GA were described. High mobility cassette 1 (HMGB1) is upregulated in metastatic prostate cancer. Using an HMGB1 knockdown DU145 cell model, HMGB1 was suggested to be directly involved in the EMT process by the CDC42/GSK-3β/SNAIL/E-cadherin signaling pathway. As an HMGB1 inhibitor, GA treatment of DU145 cells showed restrained EMT processes and disrupted cell migration [128]. In hepatocellular carcinoma cells, the aglycone of GA, 18β-glycyrrhetinic acid (**22**) reduced TGF-β-induced EMT and metastasis but not proliferation. The potential mechanism was supposed to be that GA reduced STAT3 phosphorylation [129]. A stereoisomer of GA, magnesium isoglycyrrhizinate, which is the magnesium salt of 18-α-GA, showed an inhibitory effect on EMT through the NF-κB/TWIST signaling pathway [130].

#### 3.5.2. Artemisinin (**23**)

Artemisinin is a phytochemical with a sesquiterpene lactone structure isolated from the herbal plant *Artemisia annua* by Chinese scientists that has been used as a powerful anti-malarial drug in clinics [131]. Following its discovery and utilization in clinics, a number of derivatives of artemisinin have been developed. Dihydroartemisinin (**24**) is one of the derivatives with better activity and lower toxicity. In addition to the anti-malarial effect, some other bioactivities of artemisinin and dihydroartemisinin have been developed; anti-cancer metastasis by inhibiting EMT is one of them [132]. A study reported that artemisinin reversed celecoxib-induced EMT in human ovarian epithelial adenocarcinoma SKOV3 cells. Celecoxib is a commonly used anti-inflammatory drug, but in this study it was claimed to exhibit opposite effects on the EMT process compared with artemisinin [133]. Dihydroartemisinin has been revealed to exhibit an inhibitory effect on EMT in esophageal, laryngeal, oral squamous, and breast cancer cells with different regulatory pathways. Autophagy activation associated with dihydroartemisinin exerts an anti-migration effect in esophageal cancer cells [134]. In laryngeal squamous cancer cells, dihydroartemisinin alone did not inhibit EMT and cell invasion but could block IL-6-triggered EMT and invasion by increasing the expression of miR-I30b-3p and downregulating the IL-6/STAT3/β-catenin signaling pathway [135]. In oral squamous cancer cells, dihydroartemisinin exerted a suppressive role on the EMT process by inhibiting the expression of mitochondrial calcium uniporter (MCU), which was elucidated as being upregulated in oral squamous cancer cells [136]. Dihydroartemisinin-elicited EMT inhibition in breast cancers was clarified by using canine mammary tumor cells, which are a suitable model for studies of human breast cancer research [137,138].

#### 3.5.3. Paeoniflorin (**25**)

Paeoniflorin (**25**) is a monoterpenoid glycoside isolated from the root bark of *Paeonic suffruticosa*. It has been reported to possess anti-inflammatory, immunomodulatory, and anti-cancer effects. Suppression of EMT in glioblastoma and breast cancers contributed to the anti-metastasis effects of paeoniflorin. In glioblastoma cells, c-Met was identified as a possible target of paeoniflorin for the first time. Paeoniflorin prevented EMT via K63-linked c-Met polyubiquitination-dependent autophagic degradation [139,140]. In breast cancer cells, paeoniflorin inhibited invasion and migration by suppressing hypoxia-induced EMT. Further study revealed that the PI3K/AKT pathway mediated this EMT suppression of paeoniflorin [141].

By modulating the EMT process, paeoniflorin plays a role in anti-fibrosis. In both the bleomycin-induced pulmonary fibrosis mouse model and the in vitro EMT model established in alveolar epithelial cells treated with TGF-β1, paeoniflorin effectively blocked the progression of the EMT process. The possible mechanism has been revealed as regulating a Smad-dependent pathway involving the up-regulation of Smad7 [142].

#### 3.5.4. Triptolide (**26**)

Triptolide (**26**), a tricyclic diterpenoid, is the major active component of the root of *Tripterygium wilfordii*. This phytochemical has been reported to have anti-inflammatory, proapoptotic, and tumor-repressing activities [143]. In some kinds of cancer, triptolide exerts migration inhibition effects on cancer cells, mainly through EMT blocking. In pancreatic cancer stem cells, triptolide effectively inhibited hypoxia-induced migratory activity and reversed stem-like cell features and the EMT process. Hypoxia-induced NF-κB activation was blocked concurrently and was considered the potential mechanism of triptolide in this situation [144,145]. The invasion and migration of non-small cell lung cancer cells were suppressed by restraining EMT by reducing β-catenin expression [146]. In glioma cells, triptolide showed a significant inhibitory effect on migration and triptolide invasion and reversed EMT progression [147]. Triptolide-induced autophagy was suggested as a possible mechanism of EMT inhibition. EMT-repressing effects induced by triptolide were also detected in lymphoma cells and colon stem cancer cells [143,148].

#### 3.5.5. Other Terpenoids

Some terpenoids were reported to modulate the EMT process with only a few documents, such as β-carotene (**27**), lutein (**28**), and taraxasterol (**29**). β-Carotene is a tetraterpenoid widely present in fruits and vegetables with some health-beneficial effects. One of its effects is to suppress cancer progression. Tobacco smoke-triggered EMT has been found to regulate early events of carcinogenesis. By using a mouse smoking model, β-carotene was proven to inhibit tobacco smoke-induced EMT in the stomach of mice by the Notch pathway, indicating the chemopreventive effect of β-carotene in tobacco smoke-associated gastric pathological alterations [149]. Lutein is an analogue of β-carotene. It was reported that lutein suppressed the EMT of breast cancer cells under hypoxic exposure. Downregulation of hairy and enhancer of split 1 (HES1) is involved in the effects of lutein [150]. Taraxasterol is one of the active components with a triterpenoid structure isolated from Dandelion. In non-small-cell lung cancer cells, taraxasterol prevented migration by interfering with EMT. Modulating the immune microenvironment might be one of the possible mechanisms of the anti-cancer effects of taraxasterol [151].

### 3.6. Alkaloids

**Figure 8:**
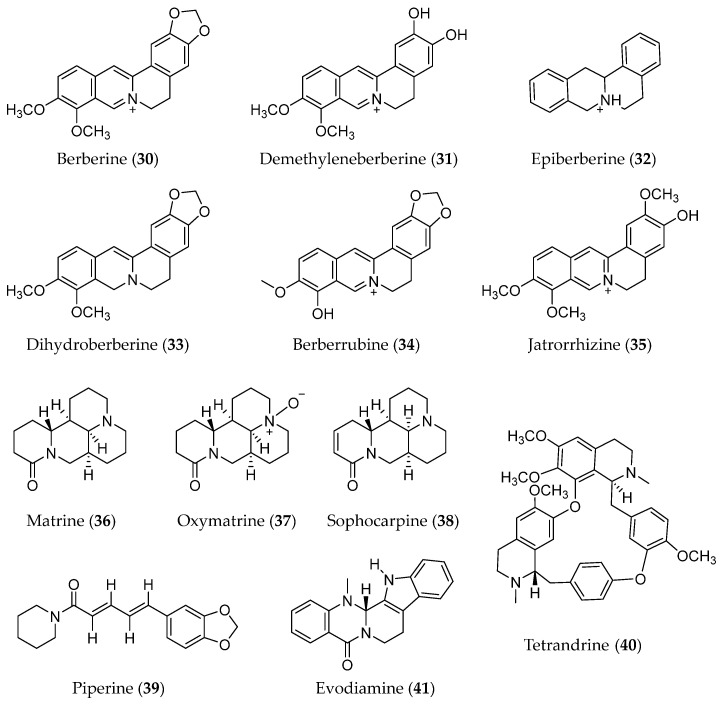
Structure of Alkaloids with EMT inhibitory effects.

Alkaloids are a class of nitrogen-containing phytochemicals with one or more nitrogen atoms in their structure. The diversity of structure and bioactivities is a characteristic of alkaloids [152,153]. In terms of anti-cancer effects, some alkaloids, including berberine (**30**) and its analogues and matrine (**36**) and its analogues (Figure 8), showed anti-metastasis effects by inhibiting the EMT process in various cancers.

#### 3.6.1. Berberine (**30**)

Berberine (**30**), an isquinoline alkaloid, is the main active component of the medicinal plants *Phellodendron amurense* and *Coptidis Rhizoma* [154]. As a commercial drug in China, the well-known pharmacological effect of berberine is as an antibacterial drug. Otherwise, anti-cancer, cardiac protection, anti-diabetes, hypolipidemic, neuroprotective, and liver-protective roles elicited by berberine have been reported [155]. Anti-metastasis is an important aspect of the anti-cancer effect of berberine. Suppressing the EMT process to block metastasis has been revealed in berberine-treated lung [156], gastric [157], cervical [158], nasopharyngeal [159], and osteosarcoma cancer cells [160]. Similar to other phytochemicals, the possible mechanisms of berberine in regulating EMT focused on some common pathways, such as TGF-β/Smad.

Some analogues of berberine also showed an EMT-suppressing effect; for example, demethyleneberberine (**31**) inhibited migration and the EMT process in colon and lung cancer cells [161,162]. Four berberine alkaloids, including berberine, epiberberine (**32**), dihydroberberine (**33**), and berberrubine (**34**), showed inhibitory effects on the migration and invasion of two kinds of breast cancer cell lines, MCF-7 and MDA-MB-231. The impact of the four berberine alkaloids on Wnt/β-catenin pathway and EMT process was different in MCF-7 and MDA-MB-231 cells. Epiberberine and berberrubine potently suppressed Wnt/β-catenin pathway and reversed EMT in MCF-7 cells. While dihydroberberine effectively inhibited Wnt/β-catenin signaling and blocked EMT in MDA-MB-231 cells. Berberine showed weak effects in both cell lines [163].

Jatrorrhizine (**35**) is another analogue of berberine; it is also derived from *Rhizoma Coptidis*. Traf2 and Nck-interacting serine protein kinase (TNIK) are cancer target proteins that are overexpressed in mammary cancer cells and contribute to the progression of cancer. Jatrorrhizine was shown to restrain TNIK-regulated Wnt/β-catenin signaling and EMT expression, which contributed to its anti-proliferation and anti-metastasis potential [164].

Blocking EMT provided an advantage for berberine to synergize with other chemotherapeutic drugs to overcome resistance [156]. In combination with gefitinib, formononetin, or even irradiation, all increased therapy sensitivity [160].

#### 3.6.2. Matrine (**35**)

Matrine, a tetracyclic quinolizidine alkaloid, is mainly extracted from Sophora plants, such as *Sophora flavescentis* and *Sophora alopecuroides* [165]. Matrine has been shown to have multiple pharmacological effects and has also been used as an anti-cancer agent [166]. Metastasis inhibition due to depression of the EMT process in cancer cells is one aspect of the anti-cancer effects of matrine. In glioma and hepatocellular carcinoma cells, matrine inhibited invasion and migration associated with the suppression of the EMT process. Reduced phosphorylation of p38 MAPK and AKT was suggested to be involved in the suppression of EMT in glioma cells [167]. While regulation of the miR-299-3p/phosphoglycerate mutase 1 (PGAM1) axis related to the constrained EMT was elicited by matrine in hepatocellular carcinoma cells [168,169].

Generally, oxymatrine (**37**) co-exists with matrine in the same plant. The two compounds have extremely similar structures and could be transformed into each other. Oxymatrine also showed inhibition of EMT in some kinds of cancer cells, such as colorectal and breast cancer cells. In colorectal cancer cells, oxymatrine inhibited EMT through TGF-β1/Smad and NF-κB pathways [170,171], and in breast cancer cells, oxymatrine suppressed the EMT process via the depressing α_V_β_3_ integrin/FAK/PI3K/AKT signaling pathway [172].

Sophocarpine (**38**) is another analogue of matrine with an extra double bond in the structure. In hepatocellular carcinoma cells, sophocarpine not only reduced cell proliferation and reversed cell malignant phenotype but also reduced the ratio of cancer stem cells. In this study, downregulation of g AKT/GSK-3β/β-catenin axis and inhibition of EMT induced by TGF-β were clarified as the mechanisms of sophocarpine [173].

Normally, when matrine and oxymatrine were used in combination with chemotherapeutic agents such as cisplatin, synergistic effects would occur due to the constrained EMT effects, and the reversing resistance effect was given more attention in these studies [174]. However, there was a completely different study: chronic oxymatrine treatment induced resistance in colon cancer cells. Resistant cells showed an EMT phenotype, and the LncRNA MALAT1 was suggested as the stimulator of oxymatrine-induced resistance [175].

#### 3.6.3. Other Alkaloids

In addition, some other alkaloids, including piperine, tetradrine, and evodiamine, have been reported to have suppressing EMT effects. Piperine (**39**) is an active ingredient in black pepper; it inhibits the EMT process in colorectal and lung cancer cells [176,177]. Tetrandrine (**40**) is a bisbenzylisoquinoline alkaloid isolated from the root of *Stephania tetrandra*. It has been proven to reverse EMT in bladder cancer cells by downregulating GLI-1 [178]. Evodiamine (**41**) is a quinazolinocarboline alkaloid that has been revealed to possess anti-metastatic ability in liver cancer Hep3B and Huh-7 cells. Modulation of the EMT process by reducing YAP levels was considered a possible mechanism for the anti-metastasis effect of evodiamine [179]. β-Carboline alkaloids were revealed as the bioactive constituents of *Arenaria kansuensis*, a plant used for the treatment of lung inflammation. A total of twelve β-carboline alkaloids all showed NF-κB/p65 pathway inhibition and EMT process reversal effects with different levels in the LPS-induced RAW264.7 inflammatory cell model and the TGF-β1 induced A549 cell model; this might be the mechanism of the antifibrogenic effect of β-carboline alkaloids [180].

## 4. Conclusions

The occurrence of EMT in cancer cells has been revealed to promote invasion, migration, and metastasis, enhance stem cell properties, and increase resistance to classical chemotherapeutics. Targeting EMT has become an attractive approach for the development of novel therapeutics to combat malignant tumors. Phytochemicals have always been the source of anti-cancer drugs. In this review, we summarized phytochemicals with EMT inhibitory effects and their modulatory mechanisms of the EMT process. There is a large batch of studies about phytochemicals with anti-EMT effects. These studies showed the value of phytochemicals as anti-EMT agents. On the other hand, the high degree of homogeneity in these studies caught our attention. A proportion of these studies even just detected typical markers of EMT and then came to the conclusion that the phytochemicals exerted their bioactivities through EMT. Under current circumstances, there is still a long way to go before converting these phytochemicals into clinical anti-cancer drugs. In order to find valuable phytochemicals with specific inhibitory effects on the EMT process, it is necessary to carry out some in-depth research, including translational studies. Meanwhile, the specific phytochemicals can be used as probes to explore novel regulatory mechanisms of the EMT process.

## Data Availability

Not applicable.
